# Effects of Phosphodiesterase 4 Inhibition on Alveolarization and Hyperoxia Toxicity in Newborn Rats

**DOI:** 10.1371/journal.pone.0003445

**Published:** 2008-10-20

**Authors:** Céline Méhats, Marie-Laure Franco-Montoya, Olivier Boucherat, Emmanuel Lopez, Thomas Schmitz, Elodie Zana, Danièle Evain-Brion, Jacques Bourbon, Christophe Delacourt, Pierre-Henri Jarreau

**Affiliations:** 1 Institut National de la Santé et de la Recherche médicale (INSERM) U767, Paris, France; 2 Université Paris Descartes, Faculté de Médecine, Paris, France; 3 INSERM U841, IMRB, équipe 06, Créteil, France; 4 Université Paris 12, Faculté de Médecine, IFR 10, Créteil, France; 5 PremUP, Paris, France; University of Giessen Lung Center, Germany

## Abstract

**Background:**

Prolonged neonatal exposure to hyperoxia is associated with high mortality, leukocyte influx in airspaces, and impaired alveolarization. Inhibitors of type 4 phosphodiesterases are potent anti-inflammatory drugs now proposed for lung disorders. The current study was undertaken to determine the effects of the prototypal phosphodiesterase-4 inhibitor rolipram on alveolar development and on hyperoxia-induced lung injury.

**Methodology/Findings:**

Rat pups were placed under hyperoxia (FiO_2_>95%) or room air from birth, and received rolipram or its diluent daily until sacrifice. Mortality rate, weight gain and parameters of lung morphometry were recorded on day 10. Differential cell count and cytokine levels in bronchoalveolar lavage and cytokine mRNA levels in whole lung were recorded on day 6.

Rolipram diminished weight gain either under air or hyperoxia. Hyperoxia induced huge mortality rate reaching 70% at day 10, which was prevented by rolipram. Leukocyte influx in bronchoalveolar lavage under hyperoxia was significantly diminished by rolipram. Hyperoxia increased transcript and protein levels of IL-6, MCP1, and osteopontin; rolipram inhibited the increase of these proteins. Alveolarization was impaired by hyperoxia and was not restored by rolipram. Under room air, rolipram-treated pups had significant decrease of Radial Alveolar Count.

**Conclusions:**

Although inhibition of phosphodiesterases 4 prevented mortality and lung inflammation induced by hyperoxia, it had no effect on alveolarization impairment, which might be accounted for by the aggressiveness of the model. The less complex structure of immature lungs of rolipram-treated pups as compared with diluent-treated pups under room air may be explained by the profound effect of PDE4 inhibition on weight gain that interfered with normal alveolarization.

## Introduction

Despite recent major advances in perinatal care, very premature infants remain prone to bronchopulmonary dysplasia (BPD), a chronic lung disease. BPD is mainly related to an arrest of lung development, characterized by minimal capillary development and fewer enlarged alveoli [1], [2]. Treatments to prevent or alleviate BPD are limited, and no currently available therapy addresses unequivocally these unmet medical needs. New therapeutic strategies are therefore necessary to maintain harmonious alveolar development and prevent BPD.

Alveolarization and distal pulmonary vascular development are intricate events that are affected by a number of insults, including prenatal or postnatal infections, inspired oxygen fraction, and mechanical ventilation [3]. A final common pathway for many of these insults is initiation and persistence of inflammation in immature lungs [4]. Increased concentrations of cytokines and leukemoid reaction have been detected in amniotic fluid and tracheal aspirate from newborns who subsequently developed BPD [5], [6]. Polymorphonuclear neutrophils invade airspaces within hours after birth and persist during the first weeks of life in the airways of these infants [7], [8]. Animal studies have demonstrated that neutrophil-induced airway inflammation promotes an arrest of alveolarization, and that inhibiting the neutrophil influx preserves alveolar development in hyperoxia-exposed newborn rats, an experimental model of BPD [9].

Elevated cAMP level suppresses the activity of immune, inflammatory, and epithelial lung cells and inhibits airway remodeling [10]. cAMP is metabolized by cyclic nucleotides phosphodiesterases (PDEs). Among the eleven families of PDEs, the PDE4 family represents the major cAMP-metabolizing enzymes in all immunocompetent cells [10], [11]. PDE4 inhibitors are active in a broad spectrum of pulmonary inflammation models and are considered as novel anti-inflammatory drugs in lung disorders [12], [13].

We therefore hypothesized that inhibition of PDE4 could prevent inflammation and hence the subsequent alveolarization impairement, and potentially oxygen-induced mortality. We used the hyperoxia model of BPD to test this hypothesis. We investigated the effect of the PDE4 selective inhibitor rolipram on airway inflammation, mortality rate, weight gain, and the extent of alveolarization assessed by morphometric methods. Inflammation was evaluated on day 6, a time when inflammation is important in this model, and assessed by differential cell count and cytokines levels in bronchoalveolar lavage (BAL) fluid and lung tissue. Alveolarization occurs between day 4 and day 14 in rat [14], and so is better evaluated in the second week of life. Due to very high mortality rate in our model, we chose to study it no later than day 10.

This issue has already been explored recently by de Visser and colleagues [15] who found that PDE4 inhibitor therapy prolonged median survival of hyperoxia-exposed pups, reduced alveolar fibrin deposition, lung inflammation as evaluated by albumin content in BAL and macrophage count in histological studies. However, possible direct effect of PDE4 inhibition on alveolar development was not evaluated in this study since no data were provided for pups treated with rolipram under room air. The present study confirms partly their data, but indicates that PDE4 inhibition presents, of its own, inhibiting effects on alveolarization.

## Results

### Assessment of inflammation and PDE4 activity at day 6

#### Inflammatory-cell count in BAL fluid

**Figure 1:**
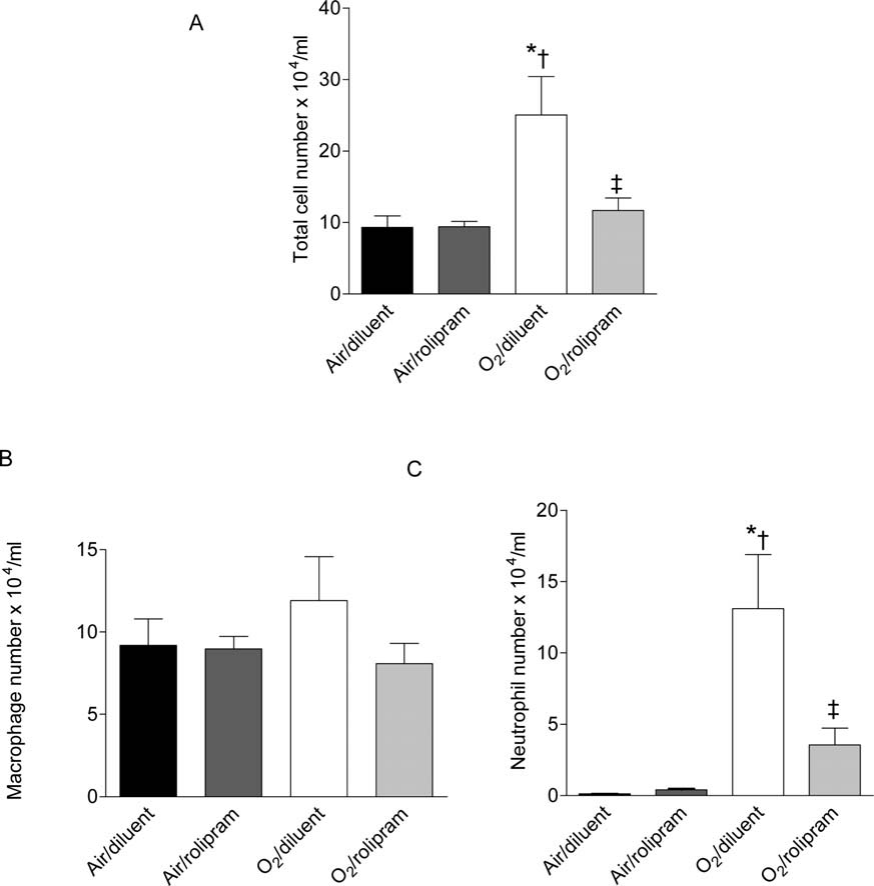
Total cell, polymorphonuclear neutrophil, and macrophage counts in BAL from rat pups exposed to hyperoxia and treated or not with rolipram. BAL fluid was collected on day 6 of life from rat pups exposed to normoxia or hyperoxia from birth and either treated with rolipram (n = 6/group) or receiving the diluent alone (littermate controls, n = 6/group). Total and differential cell counts were performed as described in Materials and Methods. Data are expressed as mean±sem. * Significantly different from the air-diluent group; † significantly different from the air-rolipram group; ‡ significant difference between oxygen-diluent and oxygen-rolipram groups.

On day 6 of life, hyperoxia increased 2.5 times the total number of inflammatory cells in BAL (ANOVA p<0.05), and induced a preferential recruitment of neutrophils that were increased 10 times as compared with control group (ANOVA p<0.001). The trend of macrophages to increase slightly was not significant (Figure 1). Rolipram had no effect on inflammatory-cell count under air condition but prevented totally the hyperoxia-induced increase in total cell number (p<0.01), and prevented partly the neutrophil increase (p<0.01).

#### Chemokine and cytokines

**Figure 2:**
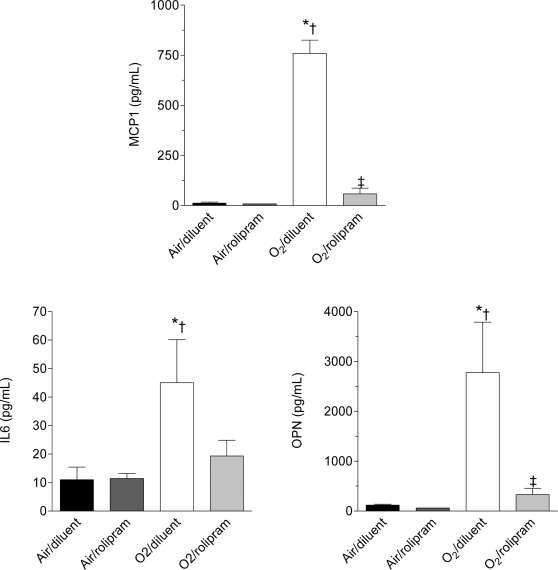
Concentration of selected chemokine (MCP1) and cytokines (IL-6 and OPN) in BAL of rat pups exposed to hyperoxia and treated or not with rolipram. Concentrations of MCP-1, IL-6, and OPN were measured by multiplex ELISA in BAL fluid, collected at day 6 of life from rat pups exposed to normoxia or hyperoxia from birth and either treated with rolipram (n = 4 or 5/group) or receiving the diluent alone (littermate controls, n = 4 or 5/group). Data are expressed as mean±sem. * Significantly different from the air-diluent group; †significantly different from the air-rolipram group; ‡ significant difference between oxygen-diluent and oxygen-rolipram groups.

**Figure 3:**
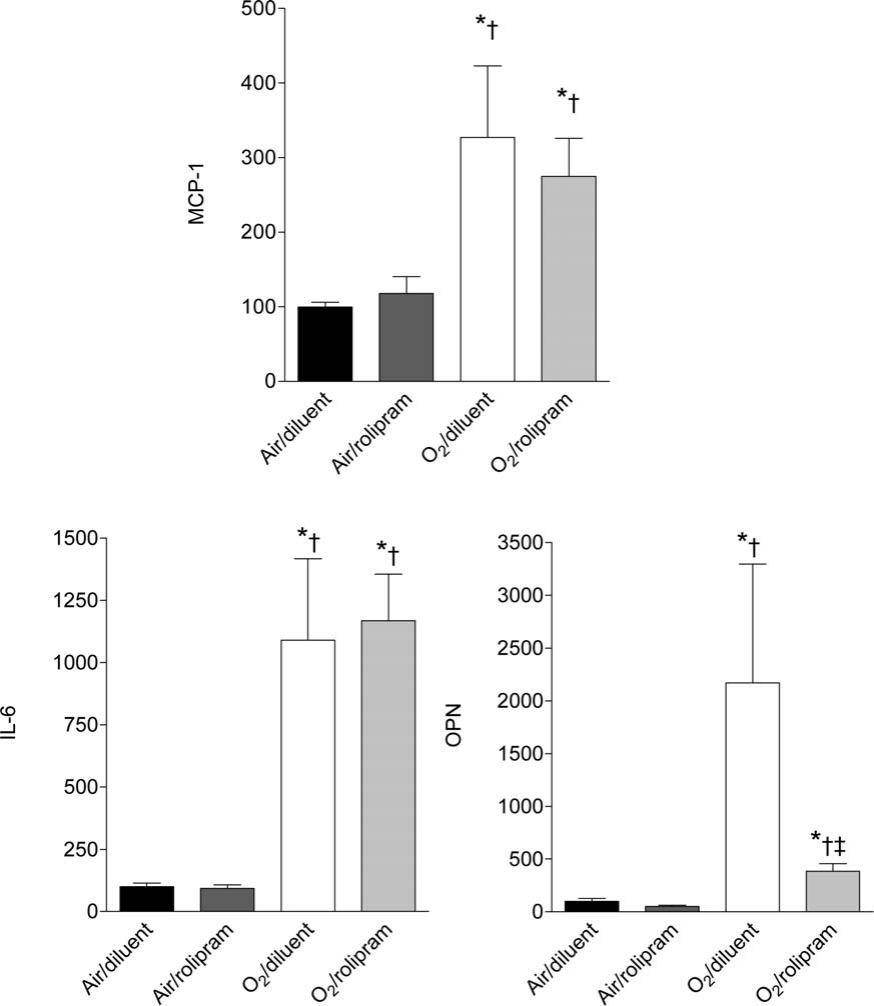
Steady-state mRNA levels of selected chemokine (MCP1) and cytokines (IL-6 and OPN) in whole lungs of rat pups exposed to hyperoxia treated with rolipram. Relative concentrations of MCP-1, IL-6, and OPN mRNAs were determined by real-time RT-PCR in whole lungs collected on day 6 of life from rat pups exposed to normoxia or hyperoxia from birth and either treated with rolipram (n = 4 or 5/group) or receiving the diluent alone (littermate controls, n = 4 or 5/group). Data are expressed as mean±sem. * Significantly different from the air-diluent group;†significantly different from the air-rolipram group; ‡ significant difference between oxygen-diluent and oxygen-rolipram groups.

Overall, the expression levels of the studied cytokines, considering either mRNAs or proteins, were different among all groups (p<0.01 for each parameter by Kruskall-Wallis analysis). On day 6 of life, hyperoxia induced a huge increase in the protein and mRNA concentrations of IL6, MCP-1, and OPN (Figures 2–3, p<0.05 to p<0.01 by Mann-Whitney U test depending of the parameter). Rolipram administration under air condition affected the protein or mRNA concentrations of none of these factors. By contrast, it prevented the increase of MCP-1 and OPN proteins under hyperoxia (p<0.01 between groups O_2_-diluent and O_2_-rolipram for both). As regards IL-6, the decrease of protein level did not reach significance (p = 0.08). Rolipram diminished the OPN mRNA level only under hyperoxia (p<0.05 between groups O_2_-rolipram and O_2_-diluent), although this was nevertheless higher than in the control group (p<0.01 between groups air-diluent and O_2_-rolipram).

#### PDE4 activity and expression

**Figure 4:**
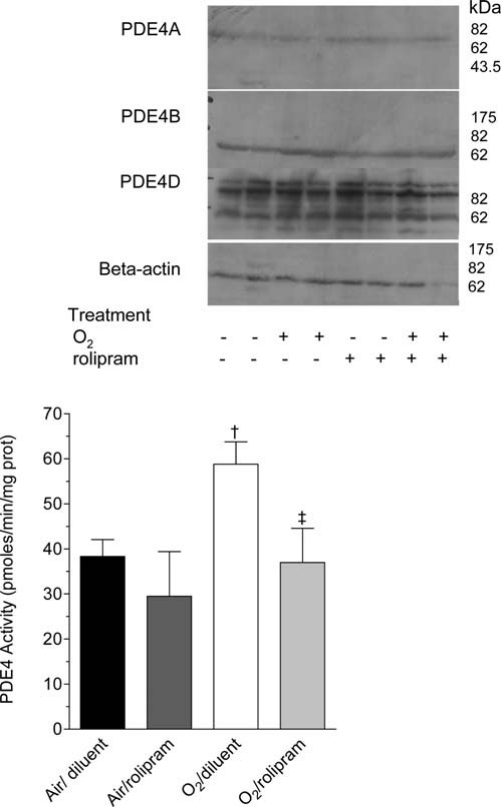
PDE4 activity in whole lung homogenates of rat pups exposed to hyperoxia and treated or not with rolipram. Rat pups exposed to normoxia or hyperoxia from birth and treated with rolipram, (n = 5/group), and their littermate controls treated with diluent alone (n = 6/group) were killed on day 6 of life. Whole lungs were dissected out and homogenized as described in Materials and Methods, and cAMP-PDE activity was measured in the absence or the presence of 10 µM rolipram. Data are expressed as mean±sem. † Significantly different from the air-rolipram group; ‡ significant difference between oxygen-diluent and oxygen-rolipram groups. Upper insert: Western blot of PDE4 proteins in whole lung of rat pups exposed to normoxia or hyperoxia from birth, and either treated with rolipram (+) or receiving the diluent alone (−); pups were killed on day 6 of life. Aliquots of lung homogenates with equivalent protein amount were subjected to 8% SDS-PAGE and immunoblotted with specific anti-PDE4A, PDE4B or PDE4D antibodies. This immunoblot is representative of 3 separate experiments with two different animals/group/experiment, 6 different animals/group. A loading control was performed with a specific anti-beta actin antibody.

On day 6 of life, whole-lung PDE4 activity was significantly different among all groups (p<0.05 by Kruskall-Wallis analysis). PDE4 activity tended to be higher in the O_2_-diluent group as compared with the air-diluent group, although this did not reach significance (p = 0.08) (Figure 4). Treatment with rolipram had no effect on PDE4 activity in normoxia, whereas it decreased it under hyperoxia (p<0.05). PDE4 family is encoded by four genes designated A through D. Whereas PDE4C is absent from adult lungs, PDE4A, 4B, and PDE4D RNAs are expressed in lungs [16]. Immunoblotting with antibodies raised against PDE4A, PDE4B, and PDE4D proteins evidenced only a slight increase of the immunosignal of a PDE4B band with an apparent molecular weight of 72 kDa (insert in Figure 4).

### Assessment of survival, growth and alveolarization at day 10

#### Survival

**Figure 5:**
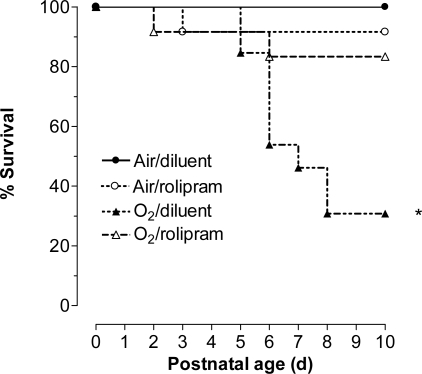
Survival of rat pups exposed to hyperoxia and treated or not with rolipram. Kaplan-Meier curve representation of survival of rat pups exposed to normoxia (room air) or hyperoxia (Fi O_2_>95%) (circles and triangles, respectively), and either treated with rolipram (n = 12/group, open symbols) or receiving the diluent alone (littermate controls, n = 13/group, closed symbols).* Significantly different from air-diluent group curve, logrank test, p = 0.017.

Overall, differences among all groups were significant (p<0.001 by Logrank). In keeping with previous studies, hyperoxia induced high mortality, mainly on days 5 and 6 of exposure, reaching 70% in the O_2_-diluent group (Figure 5). Rolipram reduced hyperoxia-induced mortality to 17%, (p<0.05 between rolipram-treated and diluent-treated groups in hyperoxia). The differences in survival between rat pups treated by rolipram under hyperoxia and those under air and receiving either rolipram or its diluent were not significant.

#### Body-weight gain

**Figure 6:**
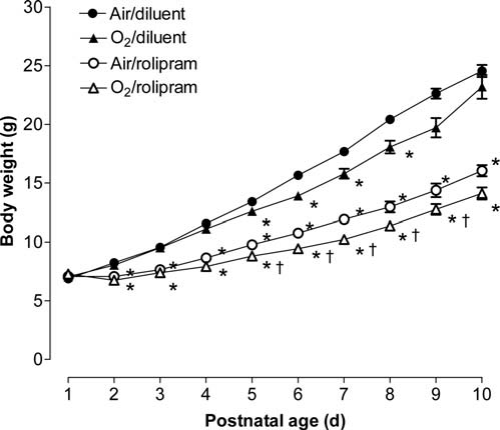
Growth of rat pups exposed to hyperoxia and treated or not with rolipram. Growth curves of rat pups exposed to normoxia or hyperoxia (circles and triangles, respectively), and either treated with rolipram (open symbols) or receiving the diluent alone (littermate controls, closed symbols). Values are mean±sem. Sixteen to 21 rat pups were included on day 0. * Significantly different from air-diluent group; † significantly different from air-rolipram group. Note that except for day 1 and day 2, all values of rolipram-treated pups were significantly lower than those of oxygen-diluent-treated pups.

Gain in body weight was different among all groups (p<0.001 by ANOVA). Hyperoxia impaired weight gain of pups all over the 10 first days of life (Figure 6). Rolipram administration decreased weight gain either under hyperoxia or normoxia in the same proportions. The decrease induced by rolipram was larger than that consecutive to hyperoxia exposure, the day-10 body weight being decreased about one third.

### Lung Morphometry

**Figure 7:**
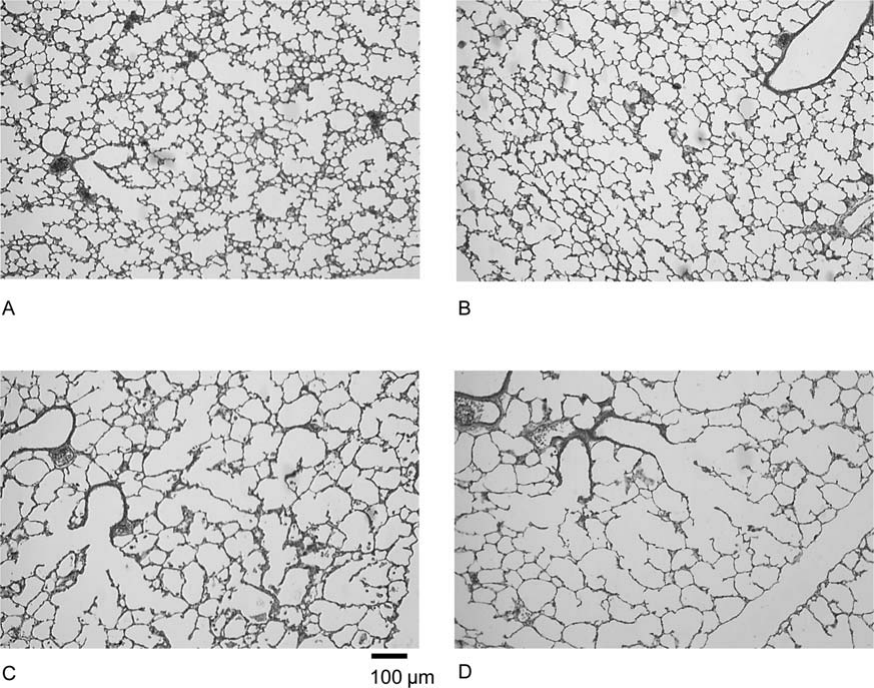
Lung histological appearance showing effects of hyperoxia and rolipram on airspace formation in rat pups. Rat pups were placed under normoxia (room air, A, B) or hyperoxia (Fi O_2_>95%, C, D) from day 0 to day 10 while receiving rolipram (B, D) or diluent (A, C). After fixation at constant pressure, lungs were embedded in paraffin, and 3 µm thick tissue slices were cut throughout the entire lung samples and stained with hematoxylin, phloxine and safran. Photographs of the alveolar region, taken at the same magnification, are presented for each treatment group (bar in C represents 50 µm). Rat pup lungs under hyperoxia exhibited a diffuse, simplified lung structure with enlarged airspaces and fewer secondary septa. Rolipram did not prevent this effect.

Gross light-microscopy evaluation of 10-day-old rat lungs suggested that hyperoxia induced enlargement of alveoli, in the diluent-treated as well as in the rolipram-treated groups (Figure 7). Alveoli appeared also underdeveloped in the air-rolipram group.

The results of morphometric analysis are summarized in Table 1.

**Table 1:** Morphometric measurements.

	AIR		HYPEROXIA	
	Diluent	rolipram	Diluent	rolipram
	(*n* = 6)	(*n* = 6)	(n = 6)	(*n* = 6)
Body weight at day 10(g)	23.5±2.3	16.6±1.7*	19.9±2.4†	12.7±1.48* † ‡
Lung volume (cm^3^)	1.26±0.17	1.12±0.10	1.01±0.22*	0.97±0.06* †
Specific lung volume (cm^3^/100 g)	5.01±0.35	6.81±0.43*	4.49±1.10†	7.15±1.05* ‡
*Alveolar surface*				
Area density (cm^2^/cm^3^)	248±15	229±30	167±30* †	145±20* †
Absolute area (cm^2^)	287±34	237±33*	162±34* †	127±13* †
Specific area (cm^2^/100 g)	1143±87	1444±196*	719±222* †	924±106* † ‡
*Alveolar Parenchyme (Vvp)*				
Volume density (%)	96±0.5	96±1.4	97±1.0	95±1.5
Absolute volume (cm^3^)	1.21±0.16	1.07±0.09	0.98±0.22*	0.92±0.06* †
Specific volume (cm^3^/100 g)	3.9±0.4	5.9±0.7*	4.3±0.5†	7.0±0.7* ‡
*Radial Alveolar Count*				
RAC (n)	10.5±0.8	6.9±1.8*	5.4±1.3*	4.5±0.6* †

Values are mean±SD.

* significantly different from the air-diluent group.

† significantly different from the air-rolipram group.

‡ significant difference between oxygen-diluent and oxygen-rolipram groups.

- *Lung volume*: overall, lung volume and specific lung volume were different among all groups (p<0.05 and p<0.01, respectively, by Kruskall-Wallis analysis). Hyperoxia decreased lung volume in pups treated by rolipram as well as in those receiving the diluent (p<0.05 for each by Mann-Whitney U test), but did not alter specific lung volume. Rolipram had no effect on absolute lung volume, but increased specific lung volume (p<0.01) either under hyperoxia or air (p<0.01).
- *Alveolar surface*: overall, alveolar-surface density, absolute and specific alveolar-surface areas were significantly different among all groups (p<0.001 for both by Kruskall-Wallis analysis). As expected, hyperoxia decreased drastically the alveolar surface density by 33%, as well as the absolute and specific surface areas by 44% and 37%, respectively (p<0.01 for rolipram- and diluent-treated pups). Rolipram had no effect on alveolar-surface area density but decreased absolute alveolar-surface area in air-exposed pups by 17% (p<0.05) and by 22%, in O_2_-exposed pups, although this effect did not reach significance (p = 0.06). Rolipram increased specific alveolar-surface area by 20% in air-exposed pups (p<0.01), and by 22% in those under hyperoxia (p<0.05) as compared to O_2_-diluent group, but this value remained lower than in groups under air (p<0.05).
- *Parenchymal alveolar volume*: overall, the differences were not significant for volume density but were significant for absolute and specific values between groups (p<0.05 and p<0.001, respectively). Absolute values were decreased by hyperoxia in both diluent (by 19%) and rolipram groups (by 14%) (p<0.05 for each), but rolipram had by itself no effect on absolute volume, neither in air nor under hyperoxia. Hyperoxia altered specific parenchymal volume neither in rolipram nor in diluent groups. Rolipram increased specific values under hyperoxia by 38% and in air by 49% (p<0.01 for each).
- *Radial alveolar count (RAC)*: overall, differences were significant for RAC among groups (p<0.01). Hyperoxia decreased significantly RAC in diluent-treated pups by 49% (p<0.01) and in rolipram-treated pups by 35% (p<0.05). Rolipram decreased RAC in air-exposed pups by 35% (p<0.01), but induced no further change in hyperoxia-exposed pups.

## Discussion

In the present study, we report that inhibition of PDE4s by rolipram in rat pups exposed to hyperoxia improved survival, decreased lung inflammation as assessed by inflammatory cell count in airspaces and cytokine measurement, but did not prevent hyperoxia-induced impairment of alveolarization. Most importantly, we evidence that rolipram presented by itself deleterious effects in normoxia, including decreased weight gain and defective alveolarization, which had not yet been reported.

Similar to our previous study [17], we studied inflammation at day 6 and alveolarization and mortality at day 10. The choice of day 6 for assessing inflammation is based on the demonstration by Deng *et al.* [18] that inflammation, as evaluated by accumulation of inflammatory cells in BAL, begins rapidly after initiating hyperoxic exposure and increases with the duration of exposure with a marked significant increase at day 6. Therefore, choosing this stage allowed accurate evaluation of inflammation and its inhibition to be performed at a time when mortality was not yet extensive. Because on the one hand alveolar septation occurs between day 4 and day 14 in the rat pup [14], and is therefore already patent on day 10, and on the other hand mortality became excessive beyond day 10, this stage was chosen to evaluate mortality rate and perform lung morphometric analysis.

Prolonged survival of rat pups under hyperoxia has previously been described with pentoxyfiline (PTX), a nonselective inhibitor of PDEs [19], and more recently with rolipram at a concentration lower than that used herein, as well as with an other PDE4 selective inhibitor, piclamilast [15]. By contrast, PTX treatment did not prolong survival in adult rats after exposure to 95% oxygen [20]. This may be explained by the difference in tolerance to oxygen between neonatal and adult animals. Rolipram is the prototypal PDE4 selective inhibitor, first described more than three decades ago [21], and it is generally admitted that at the dosage used in this study, rolipram has minimal inhibitory effect on other PDE families and does not interfere with unrelated signalling pathways [11]. A new generation of selective PDE4 inhibitors that includes piclamilast has been developed, with progress mainly in pharmacokinetics, albeit they reproduce with constancy the previously described *in vitro* and *in vivo* effects of rolipram [22], [23]. Thereby, results observed with PTX or piclamilast are certainly related to the capacity of these molecules to inhibit PDE4s. It should be pointed out that de Visser et al. [15] reported, as the result of a pilot experiment, a mortality rate of 33% for pups under hyperoxia and treated with rolipram 0.5 mg/kg/d, whereas we observed decreased mortality under hyperoxia with this dose. The choice of the latter in the present study is based on our preliminary observations that pups in room air presented high mortality rate with higher doses of rolipram, whereas minimal systemic PDE4 inhibition was achieved at doses below 0.5 mg/kg/d. Strain differences in susceptibility to oxygen and rolipram as well as those in initial weight may account for differences in findings between investigations. We indeed observed that rolipram-induced mortality was higher in thinnest pups. Since in the previous investigation [15], pups were about 2 grams lighter than those in our study on day 1, this represents a likely explanation of the different mortality rates between them.

The precise mechanisms by which PDE4 inhibition confers protection against lethal hyperoxia are still unclear. The role of PDE4s in the response to hyperoxia has been poorly studied. It has been demonstrated that hyperoxia, as well as hypoxia, increased blood level of PDEs [24] especially in young rats, but this was not correlated to mortality. We found a tendency towards higher PDE4 activity levels after hyperoxia for 6 days in lung tissue homogenates, and reported an increase of a 72 kDa PDE4B protein, presumably PDE4B2 [25]. PDE4A and PDE4D isoforms were not modified by hyperoxia. Because PDE4 activity is high and PDE4B2 is constitutively expressed in neutrophils [26], this may be related to the lung influx and sequestration of neutrophils induced by hyperoxia in the first week of life. PDE4 has been implicated in the adhesion of neutrophils to endothelial cells, their chemotaxis, and the production of oxidative burst [22]. In this view, the fact that we documented here a significant decrease in neutrophil count in BAL from rolipram-treated pups associated with a significant decrease of PDE4 activity suggests a specific role for PDE4 in oxygen toxicity. Oxygen insult drives a chronic inflammation involving free radicals, arachidonic acid metabolites, cytokines, chemokines, and recruitment and activation of neutrophils with a further production of reactive substances that react rapidly with proteins, carbohydrates and lipids, thus disrupting intercellular and intracellular homeostasis [27]. The drastic decrease of neutrophil sequestration in airspaces consecutive to rolipram treatment may thus account for its advantage for survival.

Although we did not address directly the question of oxidative stress, we determined the protein level in BAL and mRNA level in tissue of the key pro-inflammatory chemokine and cytokine MCP-1 and IL-6 to document airway inflammation. These two mediators have been shown to be elevated during hyperoxia [28], and neutralization of MCP-1 alleviated lung oxidant injury [29]. Rolipram treatment decreased significantly the accumulation of these proteins in BAL, although it did not diminish the increase of their mRNAs. This latter finding is discrepant with the study by de Visser and collaborators [15] who showed significant decrease of MCP-1 and IL-6 mRNAs on day 10 of exposure. Differences in timing of PDE4 inhibition between their and our experiments may explain this difference. The fact that these mediators were affected by rolipram only at the protein level might reflect the contribution of neutrophils and rapid turnover of these proteins with absence of accumulation in lungs. Indeed, several investigations demonstrated though a significant PDE4-inhibition effect at the transcriptional level in leukocytes [30], whereas alveolar epithelial cells concomitantly expressed cytokine mRNAs that may be poorly controlled by PDE4s in these cells.

In addition, we also determined OPN at pre- and post translational levels and showed a significant effect of rolipram on both. OPN, a secreted phosphoprotein, exists both as an immobilized ECM molecule in mineralized tissues and as a cytokine that mediates cellular functions involved in inflammation and ECM remodelling [31]. OPN gene expression is low during secondary septation, a key event of alveolarization [32], increases afterwards, and is overexpressed during neonatal hyperoxia [32] and in pulmonary fibrosis [33], the latter being a feature encountered to variable extent in BPD. Thus, PDE4 inhibition exhibits a potent anti-inflammatory effect in early postnatal period, which is consistent with other models of inflammation in the adult and with clinical trials [12], [34].

Considering the role of inflammation in the development of BPD, we therefore expected a preventive effect of rolipram on hyperoxia-induced lung injury, including its effects on alveolar development. Consistent with previous experiments using the same approach [17], alveolarization was impaired by hyperoxia, including a significant decrease in alveolar-surface area and RAC. This was the case, however, for diluent-treated as well as for rolipram-treated pups, indicating that rolipram did not restore alveolar surface area and RAC to control values. In their former study, de Visser and coworkers [15] argued for a beneficial effect of PDE4 inhibitors on lung histopathology because they found decreases in septal thickness and alveolar edema despite an absence of effect of PDE4 inhibitors on changes induced by hyperoxia in mean linear intercept. However, our more in-depth morphometric study together with the study of direct effects or rolipram in pups maintained in room air lead to modulate this interpretation. Indeed, rolipram also induced by itself a decrease of absolute alveolar-surface area and RAC in pups maintained in air. This appears suggestive of altered lung development, although this conclusion must be moderated by the observation that all specific values, i.e. when reported to body weight, were increased. Nonetheless, defective alveolarization induced by hyperoxia was not further impaired by rolipram. Neither area and volume densities, nor absolute values and RAC were significantly diminished in rolipram-treated pups under hyperoxia as compared with hyperoxia alone. It is likely that if there were pups presenting more extensively altered alveolarization, they could not survive further and were thus not evaluated.

A major gross side effect that was encountered with rolipram treatment was a significant decrease of weight gain, which is probably responsible for at least a part of altered alveolarization. Whereas pups maintained under hyperoxia showed a significant reduction of body weight gain after 5 days only as compared with those in normoxia, rolipram reduced weight gain already from the first day of injection, and its effect encompassed that of hyperoxia. This effect of rolipram was also observed, although to a lesser extent, by de Visser *et al.* [15] with half the dose used herein, even though they did not report data from a group treated with rolipram in room air. Our data allow us to conclude unequivocally to a significant effect of PDE4 inhibition independent of oxygen exposure. Rolipram may have an adverse effects on food intake most likely because of its adverse effects in the central nervous system and parietal glands that account for nausea, vomiting, and enhanced gastric acid secretion [35]. However, conversely to the previous study [15], we did observe pups nursing properly and gastric milk filling during the whole course of rolipram treatment (data not shown). Toxicological reports during preclinical studies of PDE4 inhibitors demonstrated significant inflammation of the intestinal tract and mesenteric vascularitis that suggest ill-absorption of food [36], [37]. Clinical studies with rolipram did not report weight loss in adult patients, the main adverse effects being headache, nausea, dizziness, abdominal pain, and vomiting, but it should be emphasized that the effects of PDE4 inhibitors are unknown on an unborn child, a pregnant woman, or a nursing infant. PDE4D knock-out mice, but not PDE4B or PDE4A knock-out strains, present growth retardation during their first weeks of life, catching up later in adulthood [38]. A clinical trial evaluating the effect of caffeine, which has nonselective PDE inhibitory properties and is widely used in neonatal intensive care units for preventing apnea of prematurity, induced a significant weight gain reduction in these infants, although the difference was no longer present at two years of life [39], [40]. Taken together, these observations suggest that PDE4 inhibitors may indeed interfere with statural growth in the early days of life.

In fact, impaired weight gain in rolipram-exposed pups seems to be the major factor accounting for our lung morphometric results. Massaro *et al.* [41] previously described a decrease in lung volume and absolute alveolar surface area with increased specific values in undernourished pups. The importance of nutrition in alveolar formation is known even in adult mice, and the rapid onset of genes involved in alveolar formation following refeeding after caloric restriction has been highlighted recently [42]. Therefore, it is difficult to dissociate the effect of rolipram on alveolarization from its weight-gain altering effect, although the decrease of RAC that directly reflects deficient alveolar septation argues for an additional effect independent from that on overall growth.

To conclude, PDE4 inhibition by rolipram displayed a potent inhibiting effect on hyperoxia-induced lung inflammation and mortality, but the direct inhibiting effects of the molecule on rat pup growth and lung development do not allow one to conclude favourably about its possible protective effect towards impaired lung alveolarization. Due to this side effects, it is certainly too early to propose a therapeutic role of PDE4 inhibition in the prevention of altered lung development. Nevertheless, the model of altered lung development used here is extremely aggressive, and the part of oxidant injury and inflammatory response cannot really be distinguished. Therefore, studying PDE4 inhibition in other models of arrested alveolarization, more purely inflammatory, as well as the use of a local instead of a systemic route should be considered. Moreover, the possible involvement of PDE4s in lung development is completely unknown and requires specific studies.

## Materials and Methods

Details are available as as supporting information; see Materials and Methods S1.

### Animals and hyperoxic exposure

All animal procedures were approved by our Institutional Committee on Animal Use and Care. Pregnant Sprague-Dawley rats were purchased from Charles River Laboratories (Saint-Germain sur l'Arbresle, France). Rat pups born in the laboratory and their dams were placed in chambers (Charles River) run in parallel under Fi_O2_either>95% or  = 21% (room air) as previously reported, from day 0 to day 6 or 10 [17]. The dams were exchanged daily between O_2_-exposed and room air-exposed litters to avoid lethal oxygen toxicity. Rat pups were weighed every day.

### Rolipram treatment

Rat pups received daily either an intraperitoneal injection of 0.5 mg/kg/d of rolipram (Sigma-Aldrich, St Louis, MI) or its vehicle (ethanol 0.05%), hereafter referred to as the diluent. The dose of 0.5 mg/kg/d was chosen after performing a pilot study with i.p. doses of rolipram ranging from 0.2 to 3 mg/kg/d, which showed high mortality at the highest doses and minimal systemic PDE4 inhibition at the lowest doses. This dose is twice the one used in previous study [15] in which high mortality rate was reported for 0.5 mg/k/d.

### Sample collection and bronchoalveolar lavage

Rat pups were killed by an intraperitoneal overdose of sodium pentobarbital (70 mg/kg, Ceva, Libourne, France) and were bled by aortic transsection. Lungs were either immediately lavaged, or fixed for morphometric/morphologic analysis, or dropped in liquid nitrogen and kept frozen at −80°C for further RNA extraction or protein immunoassay. BAL was performed using a total of 4 ml sterile saline; BAL fluid was centrifuged and total and differential cell counts were performed.

### Chemokine/Cytokine measurements in BAL

Measurements of MCP-1, IL-6, and osteopontin (OPN) protein concentrations were performed using the Searchlight™ multiplex sample testing by Endogen, PerbioScience (Brebieres, France).

### Determination of mRNA steady-state level in lung tissue

Total RNA was extracted using Trizol™ reagent (Invitrogen, Cergy-Pontoise, France). First-strand cDNAs were synthesized from 2 µg of RNA using the Superscript II reverse transcriptase, and random hexamer primers (Invitrogen). Real-time PCR was conducted using sequence-specific primers and “18S rRNA” as a reference with aid of ABI PRISM® 7000 Sequence Detection System (Applied Biosystems, Courtaboeuf, France).

### Phosphodiesterase activity and western blot analysis

Whole lung tissues were homogenized in hypotonic buffer. PDE activity was assayed using a modification of the Thompson and Appleman's method [43] as described previously [44]. For western blotting of PDE4, samples were boiled in Laemmli buffer, subjected to SDS-PAGE, and immunoblotted with polyclonal rabbit anti-PDE4A (AC55), polyclonal PDE4B (K118) and monoclonal PDE4D (M3S1) antibodies as previously described [45]. Correction for variations in loading was performed by blotting with an antibody raised against beta-actin (Sigma, A2066). Membranes were incubated in chemiluminescent detection reagent (ECL, GE Healthcare Life Sciences, Velizy, France), then exposed to KODAK BioMax MS film. Other sets of antibodies were used for additional controls: sheep polyclonal antibodies raised against PDE4A, PDE4B, and PDE4D [46] and gave identical results.

### Morphometry analysis

Lungs were fixed at constant pressure as described previously [47]. Alveolar surface density (Svap) was determined using point counting and mean linear intercept methods described by Weibel and Cruz-Orive [48]. Absolute surface area (Sa) per lung was calculated by multiplying surface density by lung volume. Radial alveolar count (RAC) was also performed [49], [50], [51].

### Statistical analysis

Multiple group comparisons were performed using either ANOVA or Kruskall-Wallis analysis, and two-group comparisons were made by Fishers post hoc test or Mann-Whitney U test, as appropriate. Survival was evaluated by Kaplan-Meier survival function and the logrank test. Calculations were performed with Statview® software (5.0, SAS Institute Inc, North Carolina). A P value<0.05 was considered to be statistically significant.

## Supporting Information

Materials and Methods S1 (0.05 MB DOC)
