# Supplementary material for: Effects of Phosphodiesterase 4 Inhibition on Alveolarization and Hyperoxia Toxicity in Newborn Rats
Source: PLoS One. 2008 Oct 20;3(10):e3445. doi: 10.1371/journal.pone.0003445 (PMC2563688; doi:10.1371/journal.pone.0003445)
Supplement: Materials and Methods S1 — (0.05 MB DOC) [file pone.0003445.s001.doc]

Effects of phosphodiesterase 4 inhibition on alveolarization and hyperoxia toxicity in newborn rats

# Céline Méhats, Marie-Laure Franco-Montoya, Olivier Boucherat, Emmanuel Lopez, Thomas Schmitz, Elodie Zana, Danièle Evain-Brion, Jacques Bourbon, Christophe Delacourt, Pierre‑Henri Jarreau

##### Materials and Methods S1

**Materials and Methods**

# Animals

Procedures that involved rats were approved by our Institutional Committee on Animal Use and Care and were conducted in strict accordance with guidelines for the use and care of laboratory research animals promulgated by the NIH. Pregnant Sprague-Dawley rats were purchased from Charles River Laboratories (Saint Germain sur l’Arbresle, France).

# Hyperoxic exposure

Rat pups born in the laboratory, randomly assigned to litters of equal size, and their dams were placed in Plexiglas exposure chambers (Charles River) run in parallel either with > 95% or with 21% (room air) Fi_O2_ as previously reported***,*** from day 0 to day 6 or 10 (1, 2). Food pellets and water were given ad libitum to the dams that were kept with the pups on a 12:12-h light-dark cycle. O_2_ concentrations were monitored regularly. Because adult rats have limited resistance to high O_2_, the dams were exchanged daily between O_2_-exposed and room air-exposed litters. Chambers were opened for 20 min every day to switch dams between air and O_2_ environments, to treat rat pups and to clean cages. Rat pups were weighted every day until death or sacrifice at day 6 or 10 as appropriate.

# Rolipram administration

Daily, from day 1 to day 6 or 10 of postnatal life, one half of rat pups received an intraperitoneal injection of 0.5 mg/kg/d of rolipram (Sigma-Aldrich, St Louis, MI) or its vehicle (ethanol 0.05%), hereafter referred to as the diluent.

# Sample collection

On day 6 or 10, rat pups were killed by an intraperitoneal overdose of sodium pentobarbital (70 mg/kg, Ceva, Libourne, France) and were exsanguinated by aortic transsection. Lungs were immediately lavaged, fixed for morphometric/morphologic analysis, or dropped in liquid nitrogen and kept frozen at –80°C until further RNA extraction or protein immunoassay.

**Inflammation evaluation**

**Bronchoalveolar lavage (BAL) and cell count in lung fluid**

Pups were placed in a supine position and the trachea was cannulated. Isotonic saline was gently instilled with a syringe, then withdrawn. BAL with 0.33 ml of sterile saline was performed twelve times, and the twelve lavage samples were pooled. Total cell counts were performed with a hemocytometer, then samples were centrifuged at 300x*g* for 7 min. Cell pellets were resuspended in adequate volume to obtain 10^6^ cells/ml, and differential cell counts were performed on cytospin preparations stained with Diff-Quik (Dade Behring, La Défense, France). A blinded observer counting of a minimum of 300 cells established the differential cell count. Lavaged lung tissues were discarded.

#### **Chemokine and Cytokine measurements**

# *Protein measurement in BAL*

Measurements of MCP-1, IL-6, and OPN concentrations were performed in the BAL using the Searchlight^TM^ multiplex sample testing by Endogen, PerbioScience (Brebieres, France).

# *RNA measurement in total lung*

# *RNA extraction*

Total RNA was extracted from frozen lung tissue using Trizol^TM^ reagent (Invitrogen, Cergy-Pontoise, France) according to the manufacturer’s instructions (1 ml of Trizol reagent per 50-100 mg of tissue). The quantity of RNA in each sample was determined by absorption at 260 nm (Biophotometer, Eppendorf, Hamburg, Germany). Purity of the total RNA extracted was evaluated by the 260/280 nm ratio with expected values between 1.8 and 2. Integrity and quality of each RNA sample were estimated by visualization of clear 18S and 28S ribosomal RNA bands after electrophoresis of 1 µg RNA of each sample in 1.5 % agarose gel.

# *Reverse transcription and Real-Time Quantitative PCR*

RNAs from each extraction sample were reverse-transcribed into cDNAs, using 2µg of total RNA, Superscript II reverse transcriptase, and random hexamer primers (Invitrogen) according to the supplier’s protocol. Real-time PCR was performed on an ABI Prism 7000 device (Applied Biosystems, Courtaboeuf, France) with the following protocol: initial denaturation (10 min at 95°C) and then a two-step amplification program (15 s at 95°C followed by 1 min at 60°C) repeated 40 times. Melt curve analysis was used to check that a single specific amplified product was generated. Reaction mixtures consisted of 25 ng of cDNA, SYBR Green 2 PCR Master Mix (Applied Biosystems), and forward and reverse primers in a reaction volume of 25 µl. Primers (MCP-1 forward: TGCAGGTCTCTGTCACGCTT, reverse: CTGAGACAGCACGTGGATGC, IL-6 forward: TGTTCTCAGGGAGATCTTGG, reverse: TCCAGGTAGAAACGGAACTC, OPN forward: AGAAACGGATGACTTTAAGCAAGAA, reverse: TCTCTGCATGGTCTCCATCGT) were designed with Primer Express software (Applied Biosystems). Real-time quantification was monitored by measuring the increase in fluorescence caused by the binding of SYBR Green dye to double-stranded DNA at the end of each amplification cycle. Relative expression was determined by using the Ct (threshold cycle) method of normalized samples (Ct) in relation to the expression of a calibrator sample, according to the manufacturer’s protocol. Each PCR run included a no-template control and a sample without reverse transcriptase. All measurements were performed in triplicate.

### Phosphodiesterase measurements

**Phosphodiesterase activity**

Whole lungs were homogenized in ice-cold buffer (Tris-HCl 100 mM pH 7.4, MgSO_4_ 2 mM EDTA 2mM, 10% glycerol, ß-mercaptoethanol 1 mM, and a protease inhibitor cocktail P2714 (Sigma) added freshly before use) using an all-glass homogenizer. Aliquots of the homogenates were assayed for cAMP PDE activity according to the method of Thompson and Appleman (3). PDE activities were measured with 1 µM [^3^H]-cAMP as a substrate. PDE4 activity was defined as the fraction of cAMP PDE activity inhibited by 10 µM rolipram. Protein concentrations were determined using the Bio-Rad protein assay (Bio-Rad Laboratories, Inc., Hercules, CA) with BSA as a standard.

## Western blot analysis

Samples (30 µg protein/lane) were boiled in Laemmli buffer, subjected to electrophoresis on a 8% SDS-PAGE, and blotted onto Hybond-P transfer membrane GE Healthcare-Amersham (GE Healthcare, Buckinghamshire, UK). Membranes were blocked in TBS-Tween 20 0.1% containing 5% nonfat milk. Polyclonal anti-PDE4B antibodies, donated by Dr. H. Tenor (AltanaPharma, Konstanz, Germany) were used as previously described (4). Second-step horseradish peroxidase-conjugated anti-rabbit antibodies (1:5,000) were purchased from GE Healthcare-Amersham and visualized by use of the ECL detection reagents (GE Healthcare-Amersham).

# Lung fixation and morphometry

As previously detailed (5), lungs were gently extracted and fixed with 4% paraformaldehyde through a polyethylene tracheal cannula at a constant pressure of 20 cm H_2_O. The trachea was then ligated and the lung was immersed in 4% paraformaldehyde for 24 h. Lung volumes were measured by the displacement method in the fixative solution. After fixation, lungs were embedded in paraffin and 3 µm thick tissue slices were cut throughout the entire lung samples and stained with hematoxylin, phloxine and safran. Morphometric assessment of the lung parenchymal tissue by light microscopy was performed in blind fashion on coded slides by the same operator (EZ) and repeated by a second observer (M-L F-M) to test reproducibility. A random examination of ten fields for each right and left lung was performed. Images of histologic specimens observed with the microscope (Leitz, Wetzlar, Germany) were captured by a camera (Sony, Japan) and observed on a monitor with a line grid matrix. Alveolar surface density (Svap) was measured using point counting and mean linear intercept methods described by Weibel (6). Absolute surface area (Sa) per lung was calculated by multiplying the surface density by the lung volume. Radial alveolar count was also performed (7-9). Briefly, this method consists in counting the number of distal air sacs that are transected by a line drawn from a terminal respiratory bronchiole to the nearest pleura. All the terminal bronchioles of a lung section, including all lobes were used for RAC. At least 2 RACs were performed on each lung section for each rat pup, yielding at least 10 RACs per pup.

# Statistical analysis

Values are expressed as means ± sem. Differences between three or more groups were evaluated using an ANOVA or Kruskall-Wallis test as appropriate. Differences between two groups were evaluated using a Fishers post hoc test or Mann-Whitney test as appropriate. Overall survival in relation to treatment was evaluated by Kaplan-Meier survival function and comparisons between treatment groups used the log-rank test. All calculations were performed with Statview® software (5.0, SAS Institute Inc, North Carolina). P value <0.05 was considered to be statistically significant.

# REFERENCES (for Online Data Supplement)

1. Hosford GE, Olson DM. Effects of hyperoxia on VEGF, its receptors, and HIF-2alpha in the newborn rat lung. *Am J Physiol Lung Cell Mol Physiol* 2003;285:L161-168.

2. Lopez E, Boucherat O, Franco-Montoya ML, Bourbon JR, Delacourt C, Jarreau PH. Nitric oxide donor restores lung growth factor and receptor expression in hyperoxia-exposed rat pups. *Am J Respir Cell Mol Biol* 2006;34:738-745.

3. Thompson WJ, Appleman MM. Characterization of cyclic nucleotide phosphodiesterases of rat tissues. *J Biol Chem* 1971;246:3145-3150.

4. Oger S, Mehats C, Dallot E, Ferre F, Leroy MJ. Interleukin-1beta induces phosphodiesterase 4B2 expression in human myometrial cells through a prostaglandin E2- and cyclic adenosine 3',5'-monophosphate-dependent pathway. *J Clin Endocrinol Metab* 2002;87:5524-5531.

5. Waszak P, Franco-Montoya ML, Jacob MP, Deprez I, Levame M, Lafuma C, Harf A, Delacourt C. Effect of intratracheal adenoviral vector administration on lung development in newborn rats. *Hum Gene Ther* 2002;13:1873-1885.

6. Weibel ER, Cruz-Orive LM. Morphometric methods. In: Crystal RG, West JB, Weibel ER, Barnes PJ, editors. The lung: Scientific foundations. Philadelphia, PA: Lippincott-Raven; 1997. p. 333-344.

7. Cooney TP, Thurlbeck WM. The radial alveolar count method of Emery and Mithal: A reappraisal 2--intrauterine and early postnatal lung growth. *Thorax* 1982;37:580-583.

8. Cooney TP, Thurlbeck WM. The radial alveolar count method of Emery and Mithal: A reappraisal 1--postnatal lung growth. *Thorax* 1982;37:572-579.

9. Zeltner TB, Caduff JH, Gehr P, Pfenninger J, Burri PH. The postnatal development and growth of the human lung. I. Morphometry. *Respir Physiol* 1987;67:247-267.
